# Carbon and Nitrogen Sources Have No Impact on the Organization and Composition of *Ustilago maydis* Respiratory Supercomplexes

**DOI:** 10.3390/jof7010042

**Published:** 2021-01-11

**Authors:** Deyamira Matuz-Mares, Oscar Flores-Herrera, Guadalupe Guerra-Sánchez, Lucero Romero-Aguilar, Héctor Vázquez-Meza, Genaro Matus-Ortega, Federico Martínez, Juan Pablo Pardo

**Affiliations:** 1 Departamento de Bioquímica, Facultad de Medicina, Universidad Nacional Autónoma de México, Avenida Universidad 3000, Copilco, Cd. Universitaria, Coyoacán, Ciudad de México 04510, Mexico; deya@bq.unam.mx (D.M.-M.); oflores@bq.unam.mx (O.F.-H.); lusromaguila@bq.unam.mx (L.R.-A.); hvazquez@bq.unam.mx (H.V.-M.); genaromatus@bq.unam.mx (G.M.-O.); fedem@bq.unam.mx (F.M.); 2 Laboratorio de Bioquímica y Biotecnología de Hongos, Departamento de Microbiología, Escuela Nacional de Ciencias Biológicas, Instituto Politécnico Nacional, Carpio y Plan de Ayala S/N, Miguel Hidalgo, Ciudad de México 11350, Mexico

**Keywords:** respiratory complexes, mitochondrial supercomplexes, *Ustilago maydis* mitochondria

## Abstract

Respiratory supercomplexes are found in mitochondria of eukaryotic cells and some bacteria. A hypothetical role of these supercomplexes is electron channeling, which in principle should increase the respiratory chain efficiency and ATP synthesis. In addition to the four classic respiratory complexes and the ATP synthase, *U. maydis* mitochondria contain three type II NADH dehydrogenases (NADH for reduced nicotinamide adenine dinucleotide) and the alternative oxidase. Changes in the composition of the respiratory supercomplexes due to energy requirements have been reported in certain organisms. In this study, we addressed the organization of the mitochondrial respiratory complexes in *U. maydis* under diverse energy conditions. Supercomplexes were obtained by solubilization of *U. maydis* mitochondria with digitonin and separated by blue native polyacrylamide gel electrophoresis (BN-PAGE). The molecular mass of supercomplexes and their probable stoichiometries were 1200 kDa (I_1_:IV_1_), 1400 kDa (I_1_:III_2_), 1600 kDa (I_1_:III_2_:IV_1_), and 1800 kDa (I_1_:III_2_:IV_2_). Concerning the ATP synthase, approximately half of the protein is present as a dimer and half as a monomer. The distribution of respiratory supercomplexes was the same in all growth conditions. We did not find evidence for the association of complex II and the alternative NADH dehydrogenases with other respiratory complexes.

## 1. Introduction

*Ustilago maydis* is an aerobic organism that fully depends on oxidative phosphorylation for the supply of ATP, pointing to mitochondria as a key organelle in the physiology of this basidiomycete and a potential target to study their structure–function relationships. In previous reports from our laboratory, we described the presence of the four classic respiratory complexes (complex I or NADH:ubiquinone oxidoreductase; complex II or succinate:ubiquinone oxidoreductase; complex III or ubiquinol:cytochrome c oxidoreductase; complex IV or cytochrome c oxidase) as well as complex V or ATP synthase in *U. maydis* mitochondria [1]. Other important respiratory enzymes of this organism include three alternative NADH dehydrogenases and the alternative oxidase (AOX) [1,2,3].

In the recent years, it has been reported that respiratory complexes are assembled into supramolecular structures called supercomplexes [4]. When these structures contain complexes I, III, and IV, they are called respirasomes, because the full complement of enzymes should support the transfer of electrons from NADH to oxygen [5]. Supercomplexes with different stoichiometries are found in mitochondria from different sources [5,6,7,8]. An important breakthrough in this field is the structural elucidation of the individual complexes within the mammalian mitochondrial respirasome and the I-III_2_ supercomplex [9,10,11,12], and the *Saccharomyces cerevisiae* [13] and the *Mycobacterium smegmatis* [14] III_2_IV_2_ supercomplexes. From a functional viewpoint, at least three main roles for the respiratory supercomplexes have been proposed: electron channeling from NADH or ubiquinol to oxygen [4,15], structural stabilization of complex I [4,16], and the decrease in ROS formation [17].

Since one of the proposed tasks for the respiratory supercomplexes involves the increase in respiratory chain efficiency, the presence of supercomplexes in mitochondria might be functionally linked to the cellular ATP requirements, i.e., increasing the rate of ATP synthesis entails a higher concentration of supercomplexes. The hypothesis that the type of supercomplexes will depend on the energy state of the cells is a plausible one. Based on this possibility, in this work we investigated the change in the type of supercomplexes in *U. maydis* mitochondria using different carbon and nitrogen sources in the culture media to modify the cell growth rates and thus the needs of ATP. Our results show that the type of respiratory supercomplexes was the same in all growth conditions.

## 2. Materials and Methods

### 2.1. Cell Culture

*U. maydis* cells (strain FB2) were streaked on a solid Yeast extract-Peptone-Dextrose (YPD) medium (1% glucose, 1% yeast extract, 0.25% Bacto Peptone, 2% agar) from a −70 °C glycerol stock suspension, grown at 28 °C for 12–18 h, and kept at 4 °C for no more than one week. From this solid culture, an inoculum was used to grow cells in 100 mL of a liquid YPD medium (0.5% yeast extract, 0.25% Bacto Peptone, and 0.5% glucose) for 20–24 h at 28 °C. The cells were recovered by centrifugation (1000× *g*, 10 min, 4 °C) and washed twice with sterile distilled water. Then, an aliquot of this suspension was transferred to 1 L of YPD or the minimal medium (MM) such that an optical density of 0.03 at 600 nm was obtained, and the cells were cultured for 24 h at 28 °C and 180 rpm. The YPD medium contained 1% glucose, 1% yeast extract, 0.25% Bacto Peptone. One liter of the MM was prepared by adding 62.5 mL of a salt solution made of 16 g KH_2_PO_4_, 4 g Na_2_SO_4_, 8 g KCl, 2 g MgSO_4_, 1 g CaCl_2_, and 8 mL of a trace minerals solution (60 mg H_3_BO_3_, 140 mg MnCl_2_-4(H_2_O), 400 mg ZnCl_2_, 40 mg NaMoO_4_-2(H_2_O), 100 mg FeCl_3_-6(H_2_O), and 400 mg CuSO_4_-5(H_2_O)) [18], and 3 g of (NH_4_)_2_SO_4_ as the nitrogen source; glucose, ethanol, glycerol, or lactate (all at 1%, *w*/*v*) were used as the carbon source.

### 2.2. Isolation of Mitochondria

To isolate *U. maydis* mitochondria, the method described by Sierra–Campos [3] was used with minor modifications. The cells were harvested by centrifugation at 1000× *g* for 5 min at 4 °C and washed twice with a lysis buffer (20 mM Tris-HCl, 330 mM sucrose, 2 mM ethylenediaminetetraacetic acid (EDTA), 1 mM ethylene glycol tetraacetic acid (EGTA), 100 mM KH_2_PO_4_, pH 7.4). The pellets were suspended in the lysis buffer supplemented with 0.2% bovine serum albumin (BSA), 5 mM β-mercaptoethanol, and 1 mM phenylmethylsulfonyl fluoride. A volume/wet weight ratio of 1:1 (mL/g) was used for cell resuspension. The cells were disrupted in a Mini-BeadBeater at 4 °C using glass beads with a diameter of 0.5 μm. To avoid mechanical damage of mitochondria, cell disruption was achieved by a protocol consisting of four cycles of 30 s of cell breakdown, each cycle separated by 2 min incubation in an ice bath. The cell extract was separated from the glass beads and centrifuged at 3000× *g* for 10 min at 4 °C. The supernatant was recovered and centrifuged at 12,000× *g* for 10 min at 4 °C. The mitochondrial pellets were resuspended in the lysis buffer and kept at −70 °C. Small aliquots were maintained at 4 °C for protein determination.

### 2.3. Sample Preparation for Native Electrophoresis

The respiratory complexes from *U. maydis* mitochondria were resolved by blue native PAGE as described in [19,20,21,22] with some modifications. Briefly, *U. maydis* mitochondria (2 mg protein) were suspended in 200 μL of 50 mM Bis–Tris and 500 mM 6-aminocaproic acid (pH 7.0) buffer solution and solubilized by adding 20 μL dodecyl-β-d-maltoside (DDM, 20% stock solution), corresponding to a DDM/protein ratio of 2 (g/g), or by adding 8 μL digitonin (50% stock solution), corresponding to a digitonin/protein ratio of 2 (g/g). Detergents were added drop by drop until the final weight/weight ratio was reached. The mixtures were incubated at 4 °C with gentle stirring for 30 min and centrifuged at 100,000 *g* for 35 min at 4 °C. The supernatants were recovered and, immediately before application onto linear polyacrylamide gradient gels (4–10%, 5–10%, or 3.25–7.5%), supplemented with 10 μL of a blue native buffer to a final concentration of 10% glycerol, 0.2% Coomassie Brilliant Blue G-250 and 20 mM 6-aminocaproic acid for blue native PAGE (BN-PAGE). Approximately 150 μg of protein per lane were used for electrophoresis. The anode buffer solution contained 50 mM Bis–Tris/HCl, pH 7.0; the cathode buffer solution contained 50 mM tricine, 15 mM Bis–Tris, pH 7.0; and the anionic Coomassie dye (0.02 or 0.002%). For 2D BN-PAGE, 0.02% DDM was added to the cathode buffer [22]. BN-PAGE gels were run at 4 °C and the voltage was set to 35 V for 10 h. Electrophoresis was stopped when the sharp line of the dye approached the gel front. The molecular weight of the respiratory complexes and supercomplexes was determined by their electrophoretic mobility and in-gel catalytic activity using solubilized complexes of bovine heart mitochondria as the standards [23].

### 2.4. In-Gel Enzyme Activity Assays

The in-gel assays were performed as described by Jung et al. [20]. For complex I and the alternative NADH dehydrogenase, the NADH:methylthiazolyldiphenyl-tetrazolium bromide (MTT) oxidoreductase activity was assayed in 10 mL of 10 mM Tris/HCl, pH 7.4, containing 5 mg MTT and 3.75 mg NADH. After 10–25 min, the reaction was stopped using the fixing solution (50% methanol, 10% acetic acid). For the succinate:MTT oxidoreductase activity, the assay buffer solution contained 100 mM sodium succinate, 1 mg phenazine methosulfate, 4.5 mM EDTA, and 20 mg MTT in 10 mL of 50 mM K_2_HPO_4_ (pH 7.4). The assays were performed at 20–25 °C. About 10–30 min of incubation were required to develop the color and then the reaction was stopped with the fixing solution.

For complex IV, the gel strip was incubated in 10 mL of 50 mM K_2_HPO_4_ (pH 7.2) containing 10 mg diaminobenzidine and 2 mg of horse heart cytochrome c. The assays were performed at 20–25 °C. After 30–40 min of incubation in the reaction mixture, the gel was transferred to the fixing solution.

To quantify the ATP hydrolysis activity of complex V, the gel strip was incubated at 37 °C in 50 mM glycine (adjusted to pH 8.0 with triethanolamine), 10 mM MgCl_2_, 0.2% CaCl_2_, and 8 mM ATP. ATP hydrolysis was associated with the development of a white calcium phosphate precipitate. The reaction was stopped using 50% methanol for 30 min, and then the gel was transferred to water and scanned against a dark background.

### 2.5. 2D Tricine-SDS Gel Electrophoresis and Western Blot Analysis

2D tricine-SDS-polyacrylamide gel electrophoresis (2D SDS-PAGE) was performed according to Schägger et al. [19]. After native PAGE, proteins in a gel slide were separated by 2D tricine-SDS-PAGE on a 16% polyacrylamide gel under denaturing conditions. After the run, proteins were stained with Coomassie^©^ Brilliant Blue R-125.

### 2.6. Tandem Mass Spectrometry (LC/ESI-MS/MS)

The samples were subjected to electrophoresis and the bands were excised from the Coomassie-stained SDS gel, destained, reduced, carbamidomethylated, and digested with modified porcine trypsin (Promega, Madison, WI, USA). Peptide mass spectrometric analysis was carried out using a 3200 QTRAP hybrid tandem mass spectrometer (Applied Biosystems/MDS Sciex, Concord, Toronto, ON, Canada) equipped with a nanoelectrospray ion source (NanoSpray II) and a MicroIonSpray II head [24]. The instrument was coupled online to a nanoACQUITY Ultra Performance LC system supplied by Waters (Waters Corporations, Milford, MA, USA). Briefly, spectra were acquired in the automated mode using information-dependent acquisition (IDA). Precursor ions were selected in Q1 using the enhanced MS mode (EMS) as the survey scan. The EMS was followed by an enhanced resolution scan (ER) of the three most intense ions at the low speed of 250 amu/s to determine the ion charge states and then by an enhanced product ion scan (EPI). The precursor ions were fragmented by collisionally activated dissociation (CAD) in the Q2 collision cell. The fragment ions generated were captured and mass analyzed in the Q3 linear ion trap. Database searching and protein identification were performed with the MS/MS spectra datasets using the MASCOT search algorithm (version 1.6b9, Matrix Science, London, UK, available at http://www.matrixscience.com). Mass tolerances of 0.5 Da for the precursor and 0.3 Da for the fragment ion masses were used. Carbamidomethyl-cysteine was the fixed modification and one missed cleavage for trypsin was allowed. Searches were conducted using the Fungi subset of the NCBI nr database (http://www.ncbi.nih.gov). Protein identifications were accepted when at least two MS/MS spectra matched at a 95% confidence level (*p* < 0.05).

### 2.7. Determination of Protein Content

The samples were treated with 0.017% deoxycholate and precipitated with 6% trichloroacetic acid [25]. After centrifugation at 3000× *g* for 30 min at 4 °C, the protein content was determined as described by Lowry et al. [26]. Bovine serum albumin was used as the standard.

### 2.8. Materials

Analytical grade reagents were purchased from Sigma Chemical Co. (St. Louis, MO, USA), E. Merck (Darmstadt, Germany), and BioRad (Hercules, CA, USA). Strain FB2 of *U. maydis* was obtained from the American Type Cell Collection (Manassas, VA, USA).

## 3. Results

### 3.1. Solubilization of Mitochondrial Respiratory Proteins by Dodecyl-β-d-Maltoside

**Figure 1:**
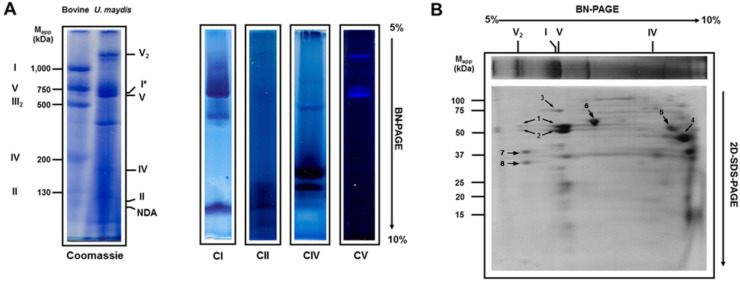
In-gel activity of *Ustilago maydis* respiratory complexes solubilized by DDM. Mitochondria were solubilized using DDM (2 g/g protein) and respiratory complexes were separated by BN-PAGE, followed by 2D SDS-PAGE. (**A**) BN-PAGE. The left panel shows the Coomassie-stained gel strips from the BN-PAGE of bovine heart and *U. maydis* mitochondrial respiratory complexes. In the right panel, CI, CII, CIV, and CV correspond to the in-gel activity assays of complexes I, II, IV, and V, respectively. Bovine heart mitochondria complexes were solubilized with DDM as described under the Materials and Methods section and used as the molecular weight standards. (**B**) For the identification of some of the respiratory chain complex subunits, proteins were resolved by 2D SDS-PAGE and analyzed by mass spectrometry. Numbers on the gel indicate the following: 1 and 2: α (UMAG_10213) and β (UMAG_10397) subunits of the ATP synthase, respectively; 3: the 75 kDa protein of complex I (UMAG_10695); 4: subunits QCR1 (UMAG_11590) and QCR2 (UMAG_01478) of complex III; 5: LPD1-dihidrolipoamide dehydrogenase (UMAG_02461) and 2-methylcitrtate dehydratase (UMAG_06344); 6: heat-shock protein HSP60 (UMAG_05831); 7: prohibitin PHB2 (UMAG_05030); 8: prohibitin PHB1 (UMAG_11092).

**Figure 2:**
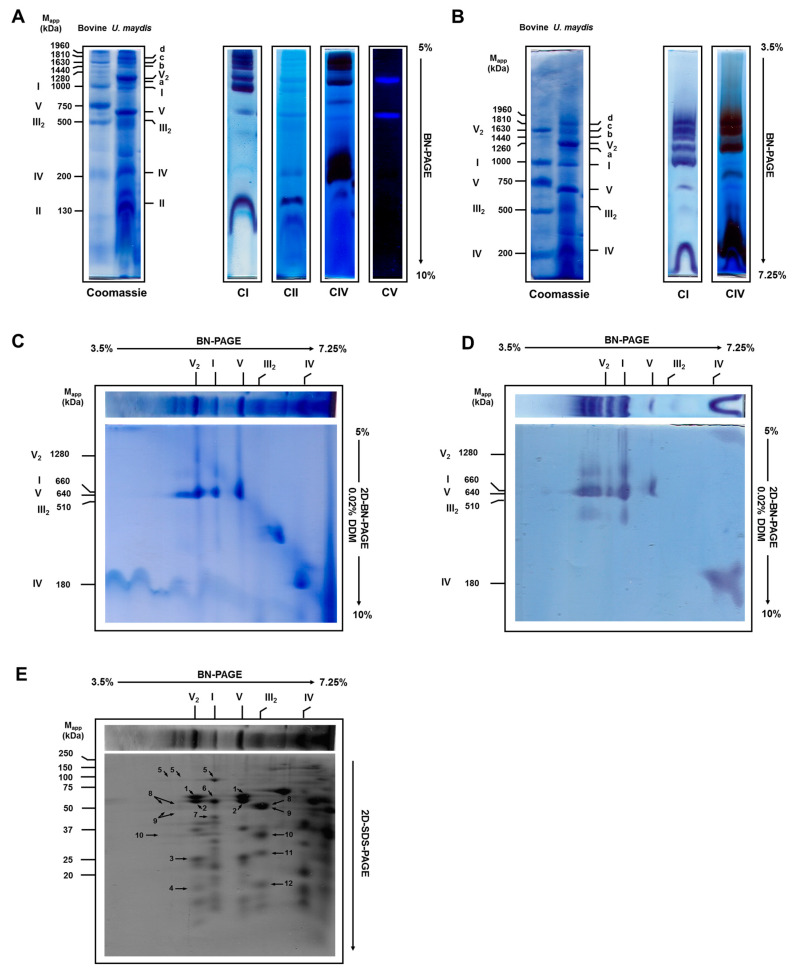
In-gel activity and identification of digitonin-solubilized mitochondrial complexes from *Ustilago maydis*. Mitochondria were solubilized using digitonin (2 g/g protein) and respiratory complexes were separated by BN-PAGE, followed by 2D BN-PAGE or 2D SDS-PAGE. (**A**) BN-PAGE was carried out on linear polyacrylamide gradient gels from 5–10%. (**A**). The left panel shows the Coomassie-stained gel strips from the BN-PAGE of bovine heart and *U. maydis* mitochondrial respiratory complexes. In the right panel, CI, CII, CIV, and CV correspond to the in-gel activity assays of complexes I, II, IV, and V, respectively. Assignment of complexes and in-gel activity assays as in Figure 1. Bovine heart mitochondria were solubilized with digitonin as described under the Materials and Methods section and used as the standard. (**B**) BN-PAGE was carried out on linear polyacrylamide gradient gels from 3.5–7.25%. (**C**) Separation of complex I from supercomplexes was carried out by 2D BN-PAGE with 0.02% DDM in the cathode buffer and the bands stained with Coomassie blue. (**D**) Separation of complex I from supercomplexes was carried out by 2D BN-PAGE with 0.02% DDM in the cathode buffer and the presence of the individual complex I was determined by NADH:MTT oxidoreductase activity. (**E**) For identification of some respiratory chain complex subunits, proteins were resolved by 2D SDS-PAGE and analyzed by tandem mass spectrometry (LC/ESI-MS/MS). The identity of the spots shown in the 2D SDS-PAGE is as follows: 1, 2, 3, and 4, the α, β, d, g and δ subunits from complex V; 5, 6, and 7, the 75, 51, and 40 kDa subunits from complex I; 8, 9, 10, 11, 12, the QCR2, QCR1, cytochrome c1, Rieske protein, and QCR7 subunits from complex III (Table 1).

When mitochondria from mammals, plants, or fungi are solubilized by dodecyl-β-d-maltoside (DDM), the respiratory complexes are observed as individual monomers, except complex III, which is a functional dimer [5,26,27]. However, when the structure of the complexes is compromised by the detergent, some domains can be detected in the gel after BN-PAGE; i.e., the F_0_F_1_ ATP synthase from bovine heart mitochondria is dissociated and the F_1_ sector migrates ahead of complex III [21]. Figure 1A shows the solubilization of *U. maydis* respiratory complexes by DDM (detergent/protein ratio of 2 g/g). Complexes I, II, IV, V, and a low molecular weight NADH dehydrogenase (NDA) were identified on the gel by their activity. The NADH dehydrogenase activity associated with complex I migrated close to 600 kDa (Figure 1A, I*), just above the ATP synthase monomer (Figure 1A) and far below its theoretical molecular mass (877 kDa, Table S1). To identify the presence of each one of the respiratory complexes, a strip from BN-PAGE was cut out and subjected to the second denaturing electrophoresis (Figure 1B). The 72 kDa band, a characteristic complex I subunit, migrates above the ATP synthase (Figure 1B). Besides, contrary to the stability of the respiratory complexes of bovine heart mitochondria solubilized by DDM, in *U. maydis*, the absence of complex III_2_ below the ATP synthase band was notorious (Figure 1B vs. Figure 2E), pointing to the high instability of this structure. As a result, subunits QCR1 and QCR2 of complex III_2_ were detected at the bottom of the gel by mass spectrometry (Figure 1B). In addition to the respiratory complexes, both the monomer (V_1_) and the dimer (V_2_) of the ATP synthase were found (Figure 1A). The molecular mass of the individual respiratory complexes of *U. maydis* was obtained using the bovine heart mitochondrial respiratory complexes as the standards [23]. Interpolation of the migration data in the standard curve gave the following results: fragment of complex I (i.e., I*), 660 kDa; complex V monomer, 640 kDa; complex IV, 180 kDa; and complex II, 100 kDa (Figure 1A).

### 3.2. Solubilization of Mitochondrial Respiratory Complexes with Digitonin

When digitonin (detergent/protein ratio of 2 g/g) was used to solubilize the respiratory complexes, the structure of complexes I and III_2_ was preserved and migrated as expected (Figure 2A). The molecular mass of complex I was around 980 kDa (Table S1), close to the predicted value obtained from its 37 subunits found in the *U. maydis* nuclear (https://fungi.ensembl.org/Ustilago_maydis/Info/Index) and mitochondrial (https://www.ncbi.nlm.nih.gov/nuccore/NC_008368.1) genomes. The proteins coded by the nuclear genome were processed by MITOPROT (https://ihg.gsf.de/ihg/mitoprot.html) to remove the mitochondrial targeting sequences [27] (Table S1). By comparison, 39 and 42 complex I subunits were reported for *Neurospora crassa* and *Yarrowia lipolytica*, respectively [28,29]. As shown in Table 1, the other single complexes migrated at about 510 kDa (complex III_2_), 640 kDa (monomeric ATP synthase), 240 kDa (cytochrome c oxidase), 140 kDa (succinate dehydrogenase), and the alternative NADH dehydrogenases (100 kDa). In these cases, the molecular weight of each complex closely agreed with the predicted one (Table 1 and Table S1).

The experimental molecular masses were determined from the BN-PAGE using the respiratory complexes of bovine heart mitochondria as the standards. The theoretical mass of each complex was obtained by searching the *U. maydis* genome (https://fungi.ensembl.org/Ustilago_maydis/Info/Index) using the amino acid sequence of the respiratory complex subunit from *S. cerevisiae* (without complex I), *N. crassa*, and *Y. lipolytica* as query sequences. The numbers inside the parentheses correspond to the numbers in Figure 2E.

Besides the individual complexes, the presence of supercomplexes containing the activities of both the NADH dehydrogenase and the cytochrome c oxidase was observed (Figure 2A). To get an insight into the number and size of the supercomplexes, BN-PAGE was carried out in 3.5–7.25% linear polyacrylamide gradient gels (Figure 2B). Above 1 MDa, three bands (a, c, and d) were associated with the NADH dehydrogenase and cytochrome c oxidase activities, while one band (b) contained only the NADH dehydrogenase activity (Figure 2B). Taking into consideration the mobility of *U. maydis* supercomplexes and the presence of NADH dehydrogenase and cytochrome c oxidase activities, the following molecular masses and stoichiometries can be proposed: a, 1200 kDa (I_1_:IV_1_); b, 1440 kDa (I_1_:III_2_); c, 1630 kDa (I_1_:III_2_:IV_1_); d, 1810 kDa (I_1_:III_2_:IV_2_) (Figure 2B and Table 2). Additionally, the dimer of complex V with a proposed molecular weight of 1260 kDa is shown.

To confirm the presence of complex I in these supercomplexes, a strip from the BN-PAGE was cut out and subjected to the second native electrophoresis with 0.02% DDM in the cathode buffer (Figure 2C). The NADH dehydrogenase activity associated with the supercomplexes was found in a fragment with a molecular mass of 660 kDa (I*) due to the dissociation of complex I by DDM (Figure 1A). The loss of complex III during the second dimension due to the presence of DDM was also evident. In addition, the absence of NADH dehydrogenase activity associated with the supercomplexes can be seen in the low molecular region of the 2D BN-PAGE gel (Figure 2D), suggesting a lack of interactions between the alternative NADH dehydrogenases and the respiratory supercomplexes. The second dimension on SDS-PAGE (Figure 2E) showed the association of the 75 kDa band (characteristic of complex I and identified by mass spectrometry) and of other bands (corresponding to subunits of complex III, QCR1, and QCR2) with supercomplexes with the NADH dehydrogenase activity (Figure 2D).

### 3.3. Respiratory Supercomplexes in Cells Growing at Different Rates

**Figure 3:**
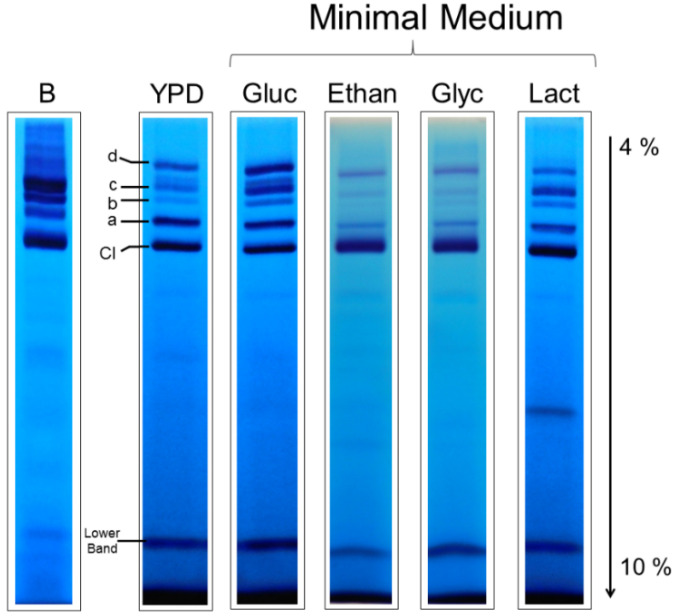
In-gel activities of complex I and the alternative NADH dehydrogenase. Mitochondria isolated from the cells grown in a rich YPD medium and the minimal media with glucose, ethanol, glycerol, or lactate were solubilized with digitonin (2 g detergent/1 g protein) and supercomplexes separated by BN-PAGE.

Based on the hypothesis that supercomplexes are responsible for higher efficiency in the electron transfer, which in turn should affect the rate of ATP synthesis, and given the fact that different proportions of complex IV were observed in *S. cerevisiae* cultured in the presence of different oxygen concentrations [30], we studied the effect of the carbon source on the composition of supercomplexes in *U. maydis* based on the fact that the growth rates—and thus the need for ATP—can change at least an order of magnitude among the selected conditions [31]. Duplication times in YPD and MM-glucose-NH_4_^+^ are around 2 h, 4 h in MM-etnanol-NH_4_^+^, and in MM-glycerol-NH_4_^+^ and MM-lactate-NH_4_^+^, duplication times are about 20 and 30 h, respectively [31]. Figure 3 shows that the same types of supercomplexes were found in the cells growing in glucose, ethanol, glycerol, or lactate despite the large differences in growth rates.

However, the relative amount of NADH dehydrogenase activity in each supercomplex with respect to the activity of free complex I changed with the growth conditions (Figure 3). For example, in the YPD medium, band a showed the highest NADH dehydrogenase activity after that of complex I, but in the glucose MM, similar activities were found for bands a, c, and d. Lower activities of supercomplexes and a major proportion of free complex I were observed with ethanol, glycerol, and lactate. It is worth noting that the band with the smallest NADH dehydrogenase activity (band b) is the one lacking complex IV activity (Figure 3). Based on the Coomassie staining, it seems that the specific NADH:MTT oxidoreductase activity (per complex I) in supercomplexes is higher than in complex I, which is in agreement with a recent report [32].

## 4. Discussion

It has been reported that the exponential phase of *U. maydis* cells growing in a YPD medium occurs in the first 10–12 h, while the stationary phase was reached at 24 h [31]. Similar growth curves were observed in either the glucose MM or the ethanol MM with ammonium sulfate as the nitrogen source; however, when glycerol or lactate was used as the carbon source, the exponential and stationary phases were reached at about 50 and 150 h, respectively [31]. Duplication times in the media containing glucose were about 2–3 h, while in glycerol and lactate, they increased to 20–30 h [31]. Interestingly, there was a change in AOX activity along the growth curve in *U. maydis* cultured in the presence of glucose, which was characterized by the absence of AOX during the exponential phase and its presence in the stationary phase [31]. These data raise the possibility of changes in the composition of the mitochondrial respiratory chain of yeast cells in the different growth media. These changes can be easily studied by solubilization of the mitochondrial proteins with DDM or digitonin, followed by the separation of the respiratory complexes using BN-PAGE.

### 4.1. The Respiratory Complexes in Ustilago maydis

Although DDM solubilized the individual respiratory complexes and the ATP synthase, some complexes were unstable under these conditions. The 660 kDa band displayed the NADH dehydrogenase activity associated with complex I. Since the theoretical molecular mass of complex I is around 900 kDa, the result suggests that DDM dissociates complex I into at least two fragments, one containing the dehydrogenase activity (designated as I*) and the other one, probably the hydrophobic domain, which was not detected by in-gel activity. This result is in agreement with the modular construction of complex I in *N. crassa* and other organisms [33,34,35,36]. It was also notorious the absence of complex III_2_ below the ATP synthase band, pointing to the high instability of this structure in the presence of DDM. As a result of this instability, subunits QCR1 and QCR2 of complex III_2_ were detected in the bottom of the gel by mass spectrometry (Figure 1B). Although complex III_2_ structure of bovine was maintained in the presence of DDM, complex III_2_ of *P. anserine* was also dissociated by DDM [37], indicating a higher susceptibility of complex III_2_ in some fungal mitochondria as compared with mammalian mitochondria.

In contrast with bovine heart mitochondria, in which the majority of complex I is sequestered in supercomplexes [8,38], in *U. maydis* mitochondria there was a significant proportion of free complex I (Figure 2A,B, Coomassie stain). Similar observations were described for *N. crassa* [7], *Y. lipolytica* [35,39], and *Debariomyces hansenii* [40] using the BN-PAGE technique. Interestingly, a recent study showed that in native bovine heart mitochondrial membrane, in the absence of any detergent, approximately 40% of complex I is not involved in the formation of supercomplexes [39], a result that diverges from the conclusions obtained by BN-PAGE experiments. Similar results were observed in native mitochondria of the yeast *Y. lipolytica* and the plant *Asparagus officinalis* [39], which agrees with the results obtained with the BN-PAGE technique. Therefore, our result suggests that the formation of respiratory supercomplexes in *U. maydis* mitochondria is not involved in the stabilization of complex I as reported for some mammalian cells [16]. Furthermore, the presence of a large proportion of free complex I in mitochondria of respiratory organisms indicates that the putative channeling of electrons in supercomplexes is not a requisite for the normal and efficient functioning of mitochondria. As shown by Blaza et al. [41] and Fedor and Hirst [42], a strict mechanistic channeling of electrons from NADH to oxygen can be discarded given the long distance between the ubiquinone-binding sites in complexes I and III [10,12,13,43]. Instead, a kinetic advantage of supercomplexes over the individual respiratory complexes is a more realistic hypothesis. Brzezinski et al. [44] showed that the equilibration time of reduced cytochrome c with the intermembrane cytochrome c pool limits the electron transfer between complexes III and IV, and the way to increase the electron flux is by decreasing the distance between complexes III and IV [44]. In agreement with this proposal, a yeast mutant which is unable to form the III_2_:IV_2_ supercomplex shows an increase in the time for cytochrome c to diffuse between the two complexes, resulting in a low efficiency of cellular energy conversion [43]. The same argument can be applied to I_1_:III_2_:IV_2_ and the ubiquinone pool. Under some conditions, for example, high respiratory rates, full exchange of QH_2_ with the ubiquinone pool becomes kinetically limiting, which would explain some results supporting substrate channeling [11].

On the other hand, although the amount of complex I bound to supercomplexes is smaller than the amount of free complex I in *U. maydis*, the in-gel NADH dehydrogenase activity of supercomplexes is proportionally higher than that found for free complex I (Figure 2A,B). This observation suggests that the incorporation of complex I into supercomplexes might increase its catalytic efficiency, as reported by Reyes–Galindo [32]. In this sense, it has also been reported that in *U. maydis*, the dimer of F_1_F_0_-ATP synthase is eight times more active than the monomer [45]. Concerning complexes III and IV, the majority of these complexes were in the free form, pointing to an excess of complex III and cytochrome c oxidase (Figure 2A,B). Free complexes III and IV are essential for the correct functioning of mitochondria, because in fungal and plant mitochondria, there are other sites for the entrance of electrons into the ubiquinone pool. For example, the alternative NADH dehydrogenases, glyceraldehyde-3-phosphate dehydrogenase and complex II, transfer electrons from specific substrates (NADH, glycerol-3-phosphate, and succinate, respectively) into the quinone pool, which in turn directs the electrons toward complex III. It is important to mention that none of these enzymes are largely associated with the main respiratory complexes. Furthermore, activities of the external alternative NADH dehydrogenases and complex II are approximately the same or even larger than complex I activity in *U. maydis* and other fungi [1,46,47,48], making free complex III a necessity for the optimal function of mitochondria.

Two interesting findings are evident. First, the band with ATPase activity above the complex V monomer (Figure 1A) and second, the two bands with cytochrome oxidase activity (Figure 1A and Figure 2A). Dissociation of V2 complex by DDM is a likely explanation of the faint band with ATPase activity. Regarding the two complex IV bands found in *U. maydis* mitochondria, different forms of complex IV have been described in other mitochondrial systems. Ukolova et al. [49] found that *Pisum sativum* mitochondria contain three forms of complex IV, a 300 kDa form (IVa), the monomer (IVb, 200 kDa), and the dimer (IV_2_, 400 kDa). The monomer was included in IV_2_ and IVa forms [49]. It seems that IVa in *Pisum sativum* mitochondria is a monomer with additional subunits [49]. Our results suggest that there is no complex IV dimer in *U. maydis* mitochondria. Instead, one form corresponds to the monomer (around 200 kDa), and the upper band might be the monomer associated with other proteins [49,50].

### 4.2. Association of Other Elements with the Respiratory Complexes

Early reports describing the copurification of complexes II and III [51] and the enzymatic analyses of *S. cerevisiae* mutants [52] suggested a weak association between these two respiratory complexes. Additional information revealed that complex II interacts with other enzymes in *S. cerevisiae* mitochondria, like the malate dehydrogenase and citrate synthase of the Krebs cycle [53]. It has been proposed that the association of complex II with the ATP synthase, the phosphate and adenine nucleotide translocators, and an ATP-binding cassette protein confers rat liver mitochondria with the ability to transport potassium [54]. More recently, the association of complex II with various supercomplexes in BN-PAGE gels of digitonin-solubilized mouse liver mitochondria was described [55], although the proportion of complex II interacting with supercomplexes was quite small.

Similar information exists for the alternative NADH dehydrogenase. By kinetic analysis, it was shown that the external and internal NADH dehydrogenase of *S. cerevisiae* mitochondria might form independent units containing complexes III and IV [56]. Association of the external and internal NADH dehydrogenases with other proteins was observed in *S. cerevisiae* [53]. Previous work showed the association of the alternative internal NADH dehydrogenase with complexes III_2_ and IV [57], suggesting the presence of a respirasome in *S. cerevisiae*. Similarly, the association of the alternative NADH dehydrogenase with complex IV in *Y. lipolytica* was described, leading to the proposal of a supercomplex formed by the external NADH dehydrogenase, complex III, and complex IV [35]. Nevertheless, in a similar work on *Y. lipolytica* mitochondria, the association between the external NADH dehydrogenase and complex IV was not found [58]. Regarding the AOX, interactions of this enzyme with respiratory complexes have also been described [59,60].

In the present work, using an in-gel assay for complex II, we did not find any evidence of the interaction of the succinate dehydrogenase or the alternative NADH dehydrogenases with other complexes in *U. maydis* mitochondria (Figure 2A,D), either because these are weak associations disrupted by the conditions of the BN-PAGE or because there are no interactions at all.

## 5. Conclusions

Some conclusions can be drawn from the results obtained in this work. (1) Since there is a large proportion of free complex I in *U. maydis* and other fungal mitochondria, its association with complex III is not a requisite for its stabilization. (2) The number and types of respiratory supercomplexes did not change with the growth rate, suggesting a small number of assembling pathways that results in a fixed composition of supercomplexes in mitochondria, but their relative activity changed with the growth conditions, indicating a differential assembly of these supercomplexes. (3) The specific activity of NADH dehydrogenase, when compared with that of free complex I, is higher in supercomplexes.

Since the formation of supercomplexes is not related with strict electron channeling or complex I stabilization, it is important to look for other possibilities. In this sense, Fedor and Hirst [42] suggested that preferred weak interactions between the complexes may protect against non-specific aggregation in the high protein concentration of the inner mitochondrial membrane and that supercomplex formation may promote quinone diffusion in the protein-dense membrane. Another interesting possibility is that supercomplexes may promote a homogeneous distribution of complexes in the inner mitochondrial membrane.

## Figures and Tables

**Table 1:** Molecular masses of *Ustilago maydis* respiratory complexes.

Band	Experimental Molecular Mass (kDa)	Theoretical Molecular Mass (kDa)	Subunit Identity	Gene Name
I	980	877	75 kDa subunit (5) 51 kDa subunit (6) 40 kDa subunit (7)	UMAG_10695 UMAG_11170 UMAG_00381
II	139	117		
III	510	474	QCR2 (8) QCR1 (9) Cytochrome c1 (10) Rieske protein (11) QCR7 (12)	UMAG_01478 UMAG_11590 UMAG_11534 UMAG_10507 UMAG_04237
IV	240	203		
V	640	599	α subunit (1) β subunit (2) d subunit (3) g subunit (4) δ subunit (4)	UMAG_10213 UMAG_10397 UMAG_12050 UMAG_00975 UMAG_01103

**Table 2:** Molecular masses and stoichiometry of *Ustilago maydis* mitochondrial respiratory supercomplexes.

Band *	Experimental Molecular Mass (kDa)	Calculated Molecular Mass (kDa)	Suggested Stoichiometry
a	1200	1220	I_1_:IV_1_
V_2_	1260	1280	
b	1440	1490	I_1_:III_2_
c	1630	1730	I_1_:III_2_:IV_1_
d	1810	1970	I_1_:III_2_:IV_2_, I_2_

* See Figure 2A. Calculated molecular masses were obtained from experimental molecular masses of the single complexes observed in the BN-PAGE using the respiratory complexes of bovine heart mitochondria as the standards. Stoichiometry was calculated using the experimental molecular masses determined by BN-PAGE and in-gel activity of complexes I and IV. The occurrence of complex III was determined by 2D SDS-PAGE and 2D BN-PAGE.

## Data Availability

Not applicable.

## Supplementary Materials

The following are available online at https://www.mdpi.com/2309-608X/7/1/42/s1, Table S1: Subunit composition of *U. maydis* respiratory complexes.
