# Supplementary material for: Carbon and Nitrogen Sources Have No Impact on the Organization and Composition of Ustilago maydis Respiratory Supercomplexes
Source: J Fungi (Basel). 2021 Jan 11;7(1):42. doi: 10.3390/jof7010042 (PMC7827470; doi:10.3390/jof7010042)
Supplement: Supplementary file 1 [file jof-07-00042-s001.zip › Table S1.docx]

Table S1. Subunit composition of *Ustilago maydis* mitochondrial respiratory complexes and ATP synthase.
